# The Role of Open Conservation Surgery in the Era of Minimally Invasive Surgery for Hypopharyngeal Cancer

**DOI:** 10.3390/medicina59101873

**Published:** 2023-10-21

**Authors:** Jooin Bang, Oh-Hyeong Lee, Geun-Jeon Kim, Dong-Il Sun, Sang-Yeon Kim

**Affiliations:** Department of Otolaryngology-Head and Neck Surgery, Seoul St. Mary’s Hospital, College of Medicine, The Catholic University of Korea, Seoul 06591, Republic of Korea; worldji@hanmail.net (J.B.); zzangyoh@gmail.com (O.-H.L.); emelenciana@naver.com (G.-J.K.); hnsdi@catholic.ac.kr (D.-I.S.)

**Keywords:** hypopharyngeal neoplasms, laryngectomy, pre-epiglottic space, prognosis

## Abstract

*Background and Objectives:* Total laryngectomy with partial pharyngectomy is traditionally the principal curative treatment for hypopharyngeal cancer; however, conservative surgical approaches that minimize functional disability are attracting increasing interest. Thus, we evaluated the appropriateness and oncological outcomes of open conservation surgery for such patients. *Materials and Methods:* We reviewed the medical records of 49 patients who underwent vertical hemipharyngolaryngectomy from 1998 to 2018 at a single institution. *Results:* Locoregional recurrences developed in 19 patients (38.8%) and distant metastases in 6 (12.2%). Histopathologically, paraglottic space invasion was apparent in 13 patients (26.5%), pre-epiglottic space invasion in 4 (8.2%), thyroid cartilage invasion in 9 (18.4%), thyroid gland invasion in 2, perineural invasion in 11 (22.4%), and lymphovascular invasion in 35 (71.4%). The 5-year overall survival of patients who underwent open conservation surgery was comparable to that of patients who underwent total laryngectomy with partial pharyngectomy (68.7% vs. 48.4%, *p* = 0.14). Pre-epiglottic space invasion significantly decreased the 5-year disease-free survival rate after open conservation surgery (69.7% vs. 17.9%, *p* = 0.01). *Conclusions:* We found that pre-epiglottic space invasion negatively impacted disease control after open conservation surgery, emphasizing the crucial role played by a preoperative evaluation during patient selection.

## 1. Introduction

Hypopharyngeal squamous cell carcinoma is an aggressive cancer with a generally poor prognosis because it tends to be diagnosed only at advanced stages [1,2]. Traditionally, total laryngectomy with partial pharyngectomy has been the mainstay treatment for advanced cancers. However, ablative surgery followed by adjuvant treatment often results in the loss of speech [3,4]. Consequently, in recent years, efforts have been made to develop reliable but delicate methods that preserve speech function [5,6].

The concept of conservation surgery was introduced in 1960; the outcomes were as good as those of conventional radical surgery [7]. Later organ-preservation approaches included transoral laser, endoscopic, and transoral robotic surgeries [5]. In a recent study, the 5-year overall survival and disease-specific survival rates were reported as 83.2% and 94.3%, respectively, for patients with hypopharyngeal cancer who underwent transoral endoscopic surgery [8]. These findings indicate comparable oncologic outcomes to those observed in patients who underwent total laryngectomy with partial pharyngectomy. Furthermore, transoral laser surgery has demonstrated favorable functional outcomes and reduced postoperative morbidity, proving it is a safe and reliable technique [9]. 

The €nitial study on open conservation surgery in hypopharyngeal cancer was published in 1980, demonstrating superior oncologic outcomes for the partial laryngopharyngectomy group when compared to total laryngectomy with partial pharyngectomy [6]. Afterward, advances in microvascular surgery allowed the development of vertical hemipharyngolaryngectomy with radial forearm free-flap reconstruction [10]. This method involved the complete resectioning of one side of the larynx and hypopharynx, followed by reconstruction using a glass plate to restore the hypopharynx and larynx. Additionally, vocal cord reconstruction was made possible using the palmaris longus tendon. In 2010, we presented a classification system for patients who might benefit from vertical hemipharyngolaryngectomy; the oncological outcomes were favorable, with a 5-year disease-free survival rate of 64% [11]. Open conservation surgery addresses the functional concerns of patients with hypopharyngeal cancer; however, few studies have explored the oncological outcomes, particularly in those with early-stage diseases [12,13]. Few researchers have explored whether open conservation surgery effectively treats advanced hypopharyngeal cancer. 

Although the demand for open conservation surgery has decreased with the rise of endoscopic and transoral robotic approaches in recent years, it is evident that open conservation surgery remains a viable treatment option that provides an opportunity to preserve the function of the hypopharynx, even in cases of advanced hypopharyngeal cancer. Therefore, we evaluated the oncological outcomes of such surgery based on the tumor-invasion status of adjacent tissues.

## 2. Materials and Methods

### 2.1. Patients 

We retrospectively reviewed the medical records of 49 patients who underwent vertical hemipharyngolaryngectomy to treat squamous cell carcinomas of the hypopharynx between January 1998 and January 2018 at Seoul St. Mary’s Hospital of the Catholic University of Korea. We retrieved patient age, sex, smoking history, primary-tumor location, TNM classification, reconstruction methods, recurrence status, and postoperative adjuvant treatments. Tumors were staged as suggested by the 8th edition of the 2017 TNM system of the American Joint Committee on Cancer. We also retrieved survival data on 40 hypopharyngeal cancer patients who underwent total laryngectomy with partial pharyngectomy over the same period; we compared their oncological outcomes to those of the test group. The exclusion criteria were a history of previous head and neck irradiation, another primary head and neck cancer, incomplete medical records, and cancers other than histopathological squamous cell carcinomas diagnosed at the final pathological review. The primary endpoint was 5-year overall survival, and the secondary outcome was 5-year disease-free survival.

### 2.2. Surgical Procedure

All patients underwent open conservation surgery, thus vertical hemipharyngolaryngectomy, followed by free-flap reconstruction. Neck dissection was guided by nodal metastasis status. Elective neck level II, III, and IV dissections were conducted when nodal metastasis was absent. Therapeutic neck dissections of levels I to V were performed when nodal metastasis was confirmed prior to surgery. Then, with careful attention to prevent injury to the hypoglossal nerve, superior laryngeal nerve, and lingual nerve, the strap muscles (the sternohyoid and thyrohyoid muscles) were resected from the hyoid bone to the head of the sternoclavicular insertion, followed by division of the hyoid bone at the midpoint. The thyroid cartilage was incised from the midline to the cricoid level, and hypopharyngeal access was achieved via transverse pharyngotomy along the upper margin of hyoid bone. This allowed vertical resection of the epiglottis when assessing tumor extent but nonetheless ensured a safe resection margin. The incised thyroid cartilage was retracted laterally to expose the primary lesion. Next, in the posterior aspects, vertical downward resection was performed from the posterior commissure to the upper end of the cricoid cartilage. The larynx, including the tumor, was excised en bloc. Glottic reconstruction employed the palmaris longus tendon for patients who underwent radial free-flap surgery of the forearm. The type of vertical hemipharyngolaryngectomy and the reconstruction method chosen was based on tumor size and location, as described previously [14].

### 2.3. Postoperative Adjuvant Treatment

Prescription of adjuvant treatment was based on a consensus formed during a multidisciplinary meeting of otorhinolaryngology, oncology, radiation oncology, nuclear medicine, plastic surgery, radiology, and pathology experts. Patients with positive or close margins, advanced-stage disease, lymphovascular or perineural invasion, multiple lymph node metastases, or extranodal extensions on the final pathological findings were prescribed postoperative chemoradiation or radiation therapy alone. 

### 2.4. Pathological Review

**Figure 1:**
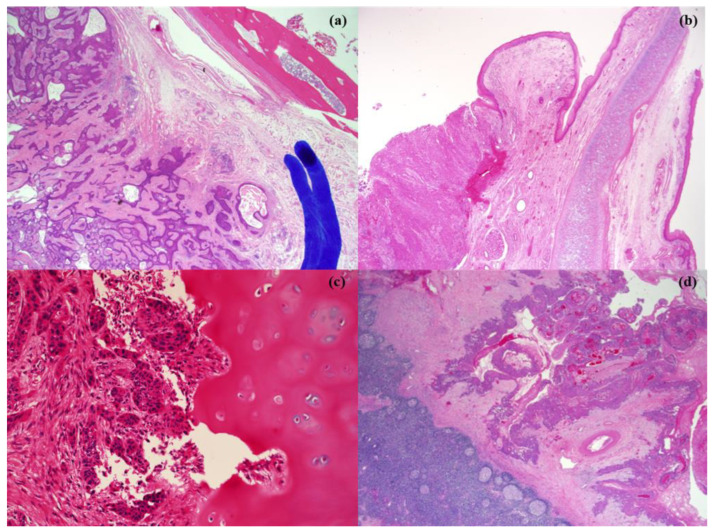
Pathologic pictures of adjacent invasion of hypopharyngeal cancer. (**a**) paraglottic space invasion, (**b**) pre-epiglottic space invasion, (**c**) thyroid cartilage invasion, and (**d**) thyroid gland invasion.

All surgical slides were meticulously examined by a single, very experienced pathologist who specializes in evaluation of head-and-neck malignancies. Serial sections were performed to determine tumor spread and invasion into adjacent structures. The staining of the preparations with hematoxylin and eosin was used as the standard method in the microscopic studies. The extent of invasion of the paraglottic and pre-epiglottic spaces, the thyroid gland, thyroid cartilage (including through-and-through extensions), and the lymphovascular and perineural invasions were thoroughly investigated (Figure 1). If the tumor involved more than two hypopharyngeal subsites, the primary site was the dominant tumor location.

### 2.5. Statistical Analysis

All statistical analyses employed SPSS ver. 25.0 software (IBM, Armonk, NY, USA). Categorical values were compared using the chi-square and Fisher’s exact tests and multiple logistic regression and correlation analyses, as appropriate. The 5-year overall survival and 5-year disease-free survival rates were derived by drawing Kaplan–Meier curves and compared using the log-rank test. A *p*-value < 0.05 was considered to indicate statistical significance.

## 3. Results

### 3.1. Patient Characteristics

Table 1 summarizes the patient characteristics. The median age was 59.8 ± 17.3 years (range 44–73 years). All 49 patients were male, and the mean follow-up time was 22.7 months. Of all patients, 38 were current smokers, 7 were ex-smokers, and 4 were non-smokers. The most common primary tumor location was the pyriform sinus (87.8%), followed by the posterior pharyngeal wall (10.2%) and postcricoid (2.0%). The pathological T classification distributions were pT1 in 6 patients (12.2%), pT2 in 21 (42.9%), pT3 in 15 (30.6%), and pT4 in 7 (14.3%). Neck nodal metastases were present in 42 patients (11 in pN1, 28 in pN2, and 3 in pN3O). Of these patients, 25 experienced failure of disease control, including 19 (38.8%) locoregional recurrences and 6 (12.2%) distant metastases. Reconstructions employed radial forearm free flaps in 48 patients (98%) and an anterolateral thigh free flap in 1 (2%). Postoperative chemoradiation was prescribed for 34 patients (69.4%), whereas 10 (20.4%) received radiation alone. Five patients (10.2%) were followed up without further treatment.

### 3.2. Histopathological Analysis 

The extent of adjacent-structure invasions and the adverse histopathological features of patients who underwent vertical hemipharyngolaryngectomy to treat hypopharyngeal cancer are listed in Table 2. The paraglottic space was the most commonly invaded in 13 patients (26.5%), followed by the thyroid cartilage in 9 (18.4%), the pre-epiglottic space in 4 (8.2%), and the thyroid gland in 2 (4.1%). Though-and-through invasion of the thyroid cartilage was not detected. Lymphovascular invasion was identified in 35 patients (71.4%), and perineural invasion was observed in 11 (22.4%).

### 3.3. Oncological Outcomes and the Histopathological Features of Patients Undergoing Vertical Hemipharyngolaryngectomy

**Figure 2:**
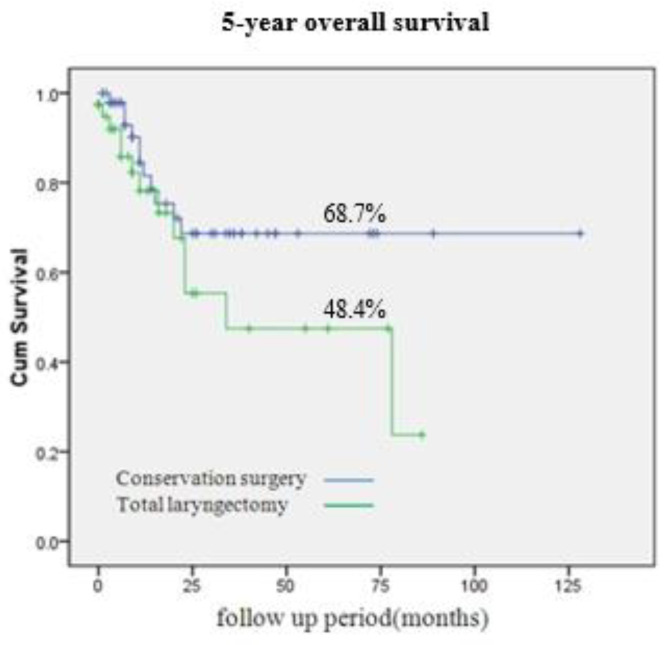
Five-year overall survival (OS) rate of hypopharynx cancer. Conservation surgery and total laryngopharygnectomy showed 68.7% and 48.4% of 5-year OS rate, respectively (*p* = 0.14).

Figure 2 compares oncological outcomes between patients who underwent total laryngectomy with partial pharyngectomy and those who had vertical hemipharyngolaryngectomy. The 5-year overall survival rate after vertical hemipharyngolaryngectomy was comparable to that after total laryngectomy with partial pharyngectomy (68.7% vs. 48.4%, *p* = 0.14). The correlation between 5-year disease-free survival and the independent histopathological parameters of patients who underwent vertical hemipharyngolaryngectomy is presented in Table 3. In univariate analysis, all evaluated histopathological parameters tended to reduce 5-year disease-free survival. Pre-epiglottic space invasion (hazard ratio [HR] = 7.30, 95% confidence interval [CI] = 0.310–11.143, *p* = 0.04) and lymphovascular invasion (HR = 1.41, 95% CI = 0.352–7.234, *p* = 0.05) were particularly significant in this context. In multivariate analysis of these two parameters, pre-epiglottic space invasion was associated with significantly decreased 5-year disease-free survival (HR = 5.35, 95% CI = 0.522–8.326, *p* = 0.04). 

### 3.4. Hypopharyngeal Cancer Prognoses According to Invasion Status of the Pre-Epiglottic and Paraglottic Spaces and Thyroid Cartilage 

**Figure 3:**
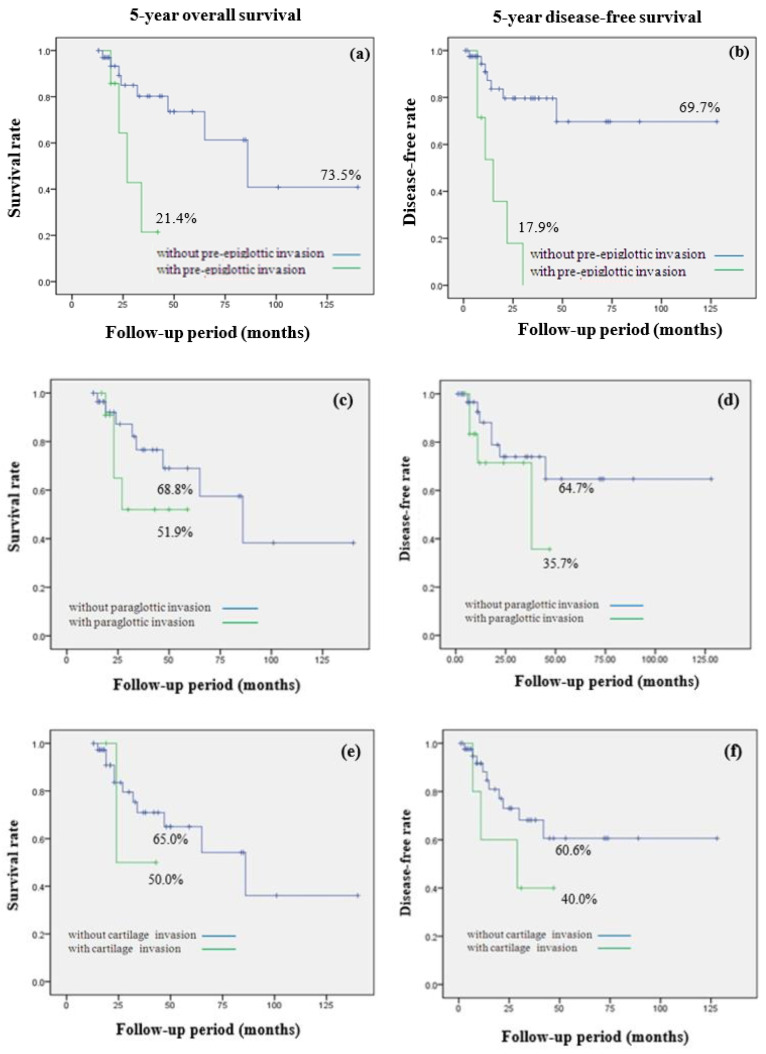
Comparison of 5-year overall survival (OS) and 5-year disease-free survival (DFS) rate in hypopharynx cancer by invasion of adjacent structures invasion. (**a**) 5-year OS; 21.4% with pre-epiglottic space invasion, 73.5% without pre-epiglottic space invasion (*p* = 0.01) (**b**) 5-year DFS; 17.9% with pre-epiglottic space invasion, 69.7% without pre-epiglottic space invasion (*p* = 0.01) (**c**) 5-year OS; 51.9% with paraglottic space invasion, 68.8% without paraglottic invasion (*p* = 0.23) (**d**) 5-year DFS; 35.7% with paraglottic space invasion, 64.7% without paraglottic space invasion (*p* = 0.24) (**e**) 5-year OS; 50.0% with thyroid cartilage invasion, 65.0% without thyroid cartilage invasion (*p* = 0.64) (**f**) 5-year DFS; 40.0% with thyroid cartilage invasion, 60.6% without thyroid cartilage invasion (*p* = 0.22).

Figure 3 compares the prognoses of hypopharyngeal cancer patients based on the invasion status of adjacent structures. A significant difference in 5-year overall survival rates was apparent between patients with and without pre-epiglottic space invasion (21.4% vs. 73.5%, *p* = 0.01). Pre-epiglottic space invasion was associated with a significantly lower 5-year disease-free survival rate (17.9% vs. 69.7%, *p* = 0.01). For patients with paraglottic space invasion, the 5-year overall survival rate was 51.9%, somewhat lower than that of patients without such invasion (68.8%), but the difference did not attain statistical significance (*p* = 0.23). Similarly, the 5-year disease-free survival rate was lower in patients with paraglottic space invasion than in those without, but the difference was not statistically significant (64.7% vs. 35.7%, *p* = 0.24). In terms of thyroid cartilage invasion (yes/no), there were no statistically significant differences in the 5-year overall survival rates (65.0% vs. 50.0%, *p* = 0.64) or the 5-year disease-free survival rates (40.0% vs. 60.6%, *p* = 0.22).

## 4. Discussion

Hypopharyngeal cancer poses significant surgical challenges, given the poor prognosis and the serious functional implications of radical procedures. Traditionally, total laryngectomy with partial pharyngectomy has been the standard surgical treatment for advanced cases of hypopharyngeal cancer since the method was introduced by the renowned surgeon Theodor Billroth in 1873 [15,16,17]. However, radical ablation often results in loss of speech and swallowing functions, significantly compromising the quality of life [4,16,18]. With advances in surgical techniques, significant efforts have been devoted to the implementation of conservative, minimally invasive surgeries; these have attracted considerable attention [8,19]. In the early 1980s, Steiner et al. developed transoral laser surgery to treat hypopharyngeal cancer; this was a major milestone [20,21]. By employing this innovative technique, it is possible to spare the structures involved in these vital functions; patients continue to communicate and eat [22]. Such preservation of functional abilities improves overall well-being and quality of life and removes any need for the permanent tracheostomy required after total laryngectomy with partial pharyngectomy [23]. Furthermore, minimally invasive surgery maintains respiratory function to some extent, improving pulmonary health and the quality of life [24]. Recently, robotic systems that aid surgery have emerged as potentially effective alternatives for the treatment of hypopharyngeal cancer. Mazerolle et al. reported that conservative, transoral robotic surgery to treat pyriform sinus cancer afforded a remarkable success rate; 96% of all patients resumed oral diets [25]. Park et al. found that transoral robotic hypopharyngectomy saving the ipsilateral arytenoid cartilage (to preserve function) allowed patient decannulation at an average of 6.3 days after surgery; the average time to the return of swallowing function in all patients was only 8.3 days [26]. We presented a mini-review of the literature, including recently published articles on Minimally Invasive Surgery for Hypopharyngeal Cancer in Table 4.

The various conservation surgeries, including transoral laser surgery, transoral robotic surgery, videolaryngoscopic surgeries, and endoscopic treatments, enhance the precision of tumor resection by affording excellent surgical visualization. This renders surgery easier by reducing the stress imposed on surgeons, in turn improving functional outcomes [18]. The continuous advances are exciting; the future of hypopharyngeal treatment is bright. However, these transoral surgeries can only be applied to early stage hypopharyngeal cancer patients. Therefore, it is necessary to evaluate the oncological outcomes of open conservation surgery, which allows for preservation of laryngeal function. Many studies have compared survival and disease-control rates after conservation surgery and conventional radical surgery [27,28]. In 1980, Ogura et al. reported that open conservation surgery to treat pyriform sinus cancers afforded better disease control than conventional radical surgery; the 3-year overall survival rate was higher (59% vs. 36%, respectively; the percentages differed significantly) [29]. A recent study found that the recurrence rates were similar in a partial laryngopharyngectomy group and a conventional radical surgery group (44% vs. 36%, *p* = 0.431) [30]. The findings consistently imply that the oncological outcomes of open conservation surgery are comparable to those of conventional radical surgery. In patients with early-stage (pT1 or pT2) hypopharyngeal cancer, in whom tumors are confined to specific subsites and thus have not extensively spread, conservation surgery may be potentially curative. Selective removal of affected areas with preservation of adjacent structures allows patients to retain essential functions, enhancing postoperative quality of life. Maintenance of both speech and swallowing function may be impossible after more-ablative surgeries. However, the utilities of conservative surgeries to treat advanced-stage hypopharangeal cancer remain unclear; efficacy and safety in such situations require more research. When tumors have infiltrated adjacent structures extensively or have metastasized to regional lymph nodes, any useful role for conservation surgery remains rather uncertain. The complexity of the disease, the risk for residual tumor cells, and the potential for disease recurrence may indicate that optimal oncological outcomes may be elusive if only conservation surgery is chosen. In our study, the pathological T classification was quite evenly distributed; almost half of all patients (44.9%) had advanced-stage disease. However, all who underwent vertical hemipharyngolaryngectomy exhibited better 5-year overall survival than those treated via total laryngectomy with partial pharyngectomy. This implies that open conservation surgery may be valuable not only for those with early-stage hypopharyngeal cancers but also for patients with advanced-stage disease, thus most patients with such cancers. However, careful patient selection is crucial to ensure that outcomes are optimal. We found that pre-epiglottic space invasion was associated with a poor prognosis, decreasing both the 5-year disease-free survival and the 5-year overall survival rates, and we believe that this finding can be utilized as valuable data in the selection of surgical candidates.

**Table 4:** Summary of demographics, prognosis, and complications.

Series	Technique	No. pts	M/F	Age, mean	OS, <2 y>, (3 y), {5 y}	DFS, <2 y>, (3 y), {5 y}	Complications
Hassid et al., 2020 [22]	TORS	22	18/4	60	(54%)	(92%)	13% bleeding
Kishimoto et al., 2020 [28]	Endo	118	114/4	65.6	(93.6%)		8% bleeding; 13% Subcutaneous emphysema
KUO et al., 2013 [5]	TLM	25	24/1	58	(79%)	(83%)	12% aspiration pneumonia; 4% Subcutaneous emphysema; 4% wound infection
Tomifuji et al., 2020 [8]	TOVS	115	106/9	67	{83.2%}	{94.3%}	2.6% bleeding
Mazerolle et al., 2015 [25]	TORS	57	52/5	60	<84%>		5% bleeding; 2% cervical hematoma; 2% pharyngostoma

No. pts: Number of patients, OS: overall survival, DFS: disease-free survival, 2 y: 2-year, 3 y: 3-year, 5 y: 5-year, TORS: trans-oral robotic surgery, Endo: Endoscopic surgery, TLM: Trans-oral laser microsurgery, TOVS: trans-oral videolaryngoscopic surgery.

There are several possible reasons why pre-epiglottic space invasion might greatly affect patients’ prognosis. The pre-epiglottic space features fibro-fatty tissue, elastic and collagen fibers, and lymphatic ducts. However, the blood supply is limited, which can trigger necrosis within a central tumor region [31,32]. This renders adjuvant treatment difficult by restricting the delivery of therapeutic agents. The lymphatic drainage pattern of hypopharyngeal cancer may explain the significance of pre-epiglottic space invasion. Lymphatic channels within the pre-epiglottic space serve as pathways for cells that will create cervical metastases; the lymphatic flow is directed toward the upper cervical lymph nodes [33]. Therefore, the presence of cancer cells within the pre-epiglottic space increases the risk for cervical metastases. Furthermore, the pre-epiglottic space is connected to the lateral paraglottic spaces via collagen-containing membranes and elastic fibers that extend from the epiglottis to the laryngeal prominence. Such connectivity implies that tumor invasion of the pre-epiglottic space indicates more advanced disease progression. Hypopharyngeal tumors typically enter the paraglottic space before invading the pre-epiglottic space [34]. Therefore, pre-epiglottic space invasion is an indicator of tumor advancement. Given the intricate anatomical relationships in play and their clinical implications, pre-epiglottic space invasion status critically affects hypopharyngeal cancer prognosis. Pre-epiglottic space involvement is a marker of necrosis, potential lymphatic dissemination, and advanced disease stage, all of which compromise patient outcomes and thus warrant careful consideration when developing treatment strategies and predicting patient prognosis.

Accurate evaluation of pre-epiglottic space invasion status is essential when planning treatment for hypopharyngeal cancer. Laryngoscopy and physical examination yield useful preliminary data but may not adequately reveal the extent of pre-epiglottic space involvement. Loevner et al. reported that unenhanced T1-weighted magnetic resonance images very sensitively revealed tumor infiltration of the pre-epiglottic space in patients with malignancies at risk for further spread; the specificity was 84%, and the sensitivity was 90% [35]. Rapoport et al. found that computed tomography reliably revealed pre-epiglottic space invasion [32]. However, current imaging techniques do not consistently detect pre-epiglottic space invasion at the microscopic level [36,37,38]. More sensitive imaging methods and/or protocols incorporating the use of complementary approaches may improve the accuracy of preoperative predictions. It is possible that positron emission tomography or other advanced imaging protocols, such as diffusion-weighted imaging, might reveal pre-epiglottic space invasion at earlier stages.

Our work had several limitations. Firstly, any retrospective study conducted at a single institution is associated with non-trivial risks of selection bias and the presence of unknown confounding factors, possibly limiting the generalizability of our findings to larger populations and/or different clinical settings. Secondly, our relatively small sample size meant that we employed compromised statistical power and precision; we may not have detected subtle differences or associations. Thirdly, we did not examine the demographic characteristics of patients who underwent total laryngectomy with partial pharyngectomy, which prevented us from analyzing the differences in parameter distribution between the two groups (total laryngectomy with partial pharyngectomy vs. wide vertical hemipharyngolaryngectomy) when assessing the prognosis. Furthermore, it is important to note that we focused on a specific form of open conservation surgery, thus not all types of conservation surgery. However, it is equally important to highlight our unique contribution. We evaluated the impacts of various histopathological features on the prognoses of hypopharyngeal cancer patients undergoing conservation surgery. Despite the aforementioned limitations, we thus offer some valuable insights that may serve as the basis for further research. These provide potential guidance to surgeons who wish to use open conservation surgery to treat patients with hypopharyngeal cancer, even those with advanced disease.

## 5. Conclusions

The prognosis of hypopharyngeal cancer patients undergoing open conservation surgery is seriously compromised when the final pathological findings reveal pre-epiglottic space invasion; both the 5-year overall survival and 5-year disease-free survival rates are reduced. It is essential to evaluate pre-epiglottic space invasion status meticulously when planning surgical treatment. Future studies should refine existing treatments and develop personalized strategies. It is essential to improve the outcomes of patients with even advanced-stage hypopharyngeal cancer scheduled for open conservation surgery.

## Figures and Tables

**Table 1:** Patient demographics.

Variables	Number of Patients (%)
All	49
Mean age, years	59.8 ± 17.3
Gender	
Men	49 (100)
Women	0 (0)
Smoking	
Current	38 (77.6)
Ex-smoker	7 (14.3)
Non-smoker	4 (8.2)
Location	
Pyriform sinus	43 (87.8)
Posterior pharyngeal wall	5 (10.2)
Postcricoid	1 (2.0)
Pathological T and N classification	
T classification	
pT1	6 (12.2)
pT2	21 (42.9)
pT3	15 (30.6)
pT4	7 (14.3)
N classification	
pN0	7 (14.3)
pN1	11 (22.4)
pN2	28 (57.1)
pN3	3 (6.1)
Reconstruction	
Radial forearm free flap	48 (98.0)
Anterolateral thigh free flap	1 (2.0)
Disease control	
Disease-free	24 (49.0)
Recurrence	25 (51.0)
Locoregional	19 (38.8)
Distant	6 (12.2)
Postoperative adjuvant treatment	
Concurrent chemoradiation	34 (69.4)
Radiation alone	10 (20.4)
None	5 (10.2)

**Table 2:** Histopathologic analysis after vertical hemipharyngolaryngectomy.

Subsites	Number of Patients (%)
Paraglottic space invasion	13 (26.5)
Pre-epiglottic space invasion	4 (8.2)
Thyroid cartilage invasion	9 (18.4)
Thyroid gland invasion	2 (4.1)
Perineural invasion	11 (22.4)
Lymphovascular invasion	35 (71.4)

**Table 3:** Univariate and multivariate analysis for 5-year disease-free survival according to the histopathological parameters.

Subsite	Univariate Analysis		Multivariate Analysis	
	Hazard Ratio (95% CI)	*p* Value	Hazard Ratio (95% CI)	*p* Value
Paraglottic space invasion	1.35 (0.289–4.493)	0.72		
Pre-epiglottic space invasion	7.30 (0.310–11.143)	0.04 *	5.35 (0.522–8.326)	0.04 *
Thyroid cartilage invasion	1.89 (0.440–4.952)	0.52		
Thyroid gland invasion	1.27 (0.125–3.315)	0.76		
Perineural invasion	3.79 (0.285–6.163)	0.95		
Lymphvascular invasion	1.41 (0.352–7.234)	0.05 *	1.58 (0.492–3.573)	0.25

CI: confidence interval, * for statistical significance.

## Data Availability

The datasets used and/or analyzed during the current study available from the corresponding author on reasonable request.
